# Exploring Parametric and Mechanistic Differences between Expi293F^TM^ and ExpiCHO-S^TM^ Cells for Transient Antibody Production Optimization

**DOI:** 10.3390/antib12030053

**Published:** 2023-08-10

**Authors:** Jing Zhou, Guoying Grace Yan, David Cluckey, Caryl Meade, Margaret Ruth, Rhady Sorm, Amy S. Tam, Sean Lim, Constantine Petridis, Laura Lin, Aaron M. D’Antona, Xiaotian Zhong

**Affiliations:** BioMedicine Design, Medicinal Sciences, Pfizer Worldwide R&D, 610 Main Street, Cambridge, MA 02139, USA; jing.zhou3@pfizer.com (J.Z.); guoying.yan@pfizer.com (G.G.Y.); dcluckey2525@gmail.com (D.C.); caryl.meade@pfizer.com (C.M.); margaret.ruth@pfizer.com (M.R.); rhady.sorm@pfizer.com (R.S.); amy.tam@pfizer.com (A.S.T.); sean.limkh@pfizer.com (S.L.); petridisc@gmail.com (C.P.); laura.lin@pfizer.com (L.L.); aaron.dantona@pfizer.com (A.M.D.)

**Keywords:** antibody production, transient gene expression, HEK cells, CHO cells, Kappa and lambda light chain, transfection reagents, DNA uptake trafficking pathways

## Abstract

Rapidly producing drug-like antibody therapeutics for lead molecule discovery and candidate optimization is typically accomplished by large-scale transient gene expression technologies (TGE) with cultivated mammalian cells. The TGE methodologies have been extensively developed over the past three decades, yet produce significantly lower yields than the stable cell line approach, facing the technical challenge of achieving universal high expression titers for a broad range of antibodies and therapeutics modalities. In this study, we explored various parameters for antibody production in the TGE cell host Expi293F^TM^ and ExpiCHO-S^TM^ with the transfection reagents ExpiFectamine^TM^ and polyethylenimine. We discovered that there are significant differences between Expi293F^TM^ and ExpiCHO-S^TM^ cells with regards to DNA complex formation time and ratio, complex formation buffers, DNA complex uptake trafficking routes, responses to dimethyl sulfoxide and cell cycle inhibitors, as well as light-chain isotype expression preferences. This investigation mechanistically dissected the TGE processes and provided a new direction for future transient antibody production optimization.

## 1. Introduction

Therapeutic antibodies are the largest class of new drugs occupying half of the global top 10 therapeutics products sold in 2022 [1]. These therapeutic interventions have achieved significant efficacies with reduced side effects in a number of disease areas especially for cancer, autoimmune, and inflammatory disorders. There is a huge and growing demand for recombinant antibodies in biopharmaceutical research and development pipelines. Expediting antibody discovery processes and shortening the development cycle are critical in effectively bringing valuable antibody drug products to the needed patients in clinics. Since antibodies are therapeutic protein drugs that are produced by cultivated mammalian cells, the antibody protein production process is an essential step for biotherapeutics research and development, e.g., lead candidate identification and optimization, in vitro and in vivo efficacy studies, pharmacokinetics, and toxicology studies, as well as clinical applications. Tremendous research efforts have been put into method development for robust transgene production over the decades.

While the chromosomal-integrated stable cell line approach is routinely utilized for generating clinical-grade recombinant antibodies, transient gene expression technologies (TGE) with episomal plasmid transfection in mammalian cells have been widely used for the rapid production of research-grade antibodies in milligrams and grams quantities [2,3,4,5,6]. Without the need for gene recombination into the host genomes which usually takes weeks to be stabilized, transient transfection of target DNAs complexed with cationic lipids, cationic polymers, or calcium phosphate, enables a fast and efficient protein scale-up production for a large number of antibody candidates in days. Mammalian cell hosts employed by the TGE technologies ensure not only functional protein folding and assembly but also proper post-translational modifications. The predominant host cell line for TGE is human embryonic kidney (HEK)-293 cells, which are known to be easy to use, highly transfectable to plasmid DNAs, and adaptable to robust high cell density growth in serum-free suspension. Another major TGE host of choice is Chinese hamster ovary (CHO) cells, the most common stable cell hosts for commercial and clinical protein production, which offers a potential advantage of end-to-end product alignment.

The TGE technologies have made a tremendous stride in improving expression titers and protein yields over the past thirty years. Through cell line engineering, vector engineering, and process engineering, antibody titers greater than 1 g/L produced transiently by HEK293 cells on a large scale have been reported [7,8]. Similarly, despite the initial modest expression yields [9,10], the transient CHO systems also reach the expression titer range of 1–3 g/L for a number of antibodies [11,12,13,14,15,16,17]. However, TGE technologies still face significant challenges. Transient expression levels of many recombinant antibody constructs remain 1–2 orders of magnitude lower than those from the corresponding stable cell lines. For some low-expression antibodies, reasonable expression titers still cannot be obtained. To meet the demands for rapid hit screenings and for increasing molecular complexities of multispecific modalities [18,19,20], the TGE workflow and protocols need constant optimization. In addition, while the above-mentioned proprietary HEK293 and CHO cell lines have significant advantages for protein production, only commercial cell lines Expi293F^TM^ and ExpiCHO-S^TM^ [8,13,21,22], are readily available to the scientific communities. Research efforts on investigating and further optimizing these highly productive cell hosts [23] are imperatively needed, especially in the areas like transfection parameters and mechanisms as well as new strategies for production enhancements. The outcomes from such studies with impacts on the production of various biotherapeutics hits should help improve the overall TGE technologies in varied systems.

In this study, we set out to fine-tune several key aspects of the transfection processes by the reductionist “one factor at a time” approach [7,9,10,11,24,25,26,27,28] in both Expi293F^TM^ and ExpiCHO-S^TM^ cells. Transfection reagents of ExpiFectamine^TM^ and polyethyleneimine (PEI) were both employed for plasmid DNA transfection. The TGE protocols with optimized parameters that can significantly boost antibody production were developed. We have uncovered that the Expi293F^TM^ and ExpiCHO-S^TM^ systems are different from each other in multiple aspects, e.g., complex formation time and volume, DNA-to-PEI ratio, responses to dimethyl sulfoxide (DMSO) and cell cycle inhibitors, as well as antibody light-chain isotype preferences. These observations should provide new insight into the mechanistic differences between the TGE in Expi293F^TM^ and ExpiCHO-S^TM^.

## 2. Materials and Methods

### 2.1. Chemicals and Reagents

DMSO (CAT#02650), sodium butyrate (CAT#156547), valproic acid sodium salt (VPA, CAT#P4543), carbenicillin disodium salt (CAT#C1389), Genestein (CAT# G6649), chlorpromazine (CAT# C8138), Filipin (CAT# F9765), and methyl-β-cyclodextrin (CAT# C4555) were purchased from Sigma-Aldrich (Burlington, MA, USA). Sodium butyrate (CAT#M19-137) was from EMD Millipore (Billerica, MA, USA). Polyethyleneimine Max powder (CAT#24765-1, Polysciences Inc., Warrington, PA, USA) was dissolved in cell culture grade sterile water, adjusted to pH 7.0, sterile-filtered to the final stock solution of 1.5 mg/mL, aliquoted and stored at −20 °C. Tryptone N1 powder (CAT#19553, Organotechnie, La Courneuve, France) was dissolved into Expi293 expression medium to a final concentration of 20% (*w*/*v*) and sterile filtered. ExpiFectamine™ 293 Transfection Kit (CAT#A14524) and ExpiFectamine^TM^ CHO Transfection Kit (CAT#A29131) were purchased from Thermo Fisher Scientific (Waltham, MA, USA).

### 2.2. Cell Culture

Expi293F™ cells (Cat#A14527) and ExpiCHO-S™ cells (CAT#A29127) were purchased from Thermo Fisher Scientific. Expi293F^TM^ expression medium (Thermo Fisher, CAT#A1435101) and ExpiCHO-S^TM^ expression media (Thermo Fisher, Cat#A2910001) were used to culture the corresponding cells in Multitron incubator shakers (INFORS HT, Bottmingen, Switzerland) or Kuhner incubator shakers (Kuhner, Birsfelden, Switzerland) with 8% CO_2_ at 37 °C. The Expi293F™ cells were routinely maintained in Thomson Optimum Growth^®^ shake flasks (Thomson, Oceanside, CA, USA) at 120 rpm and ExpiCHO-S™ Cells were cultured at 68 rpm in Erlenmeyer flasks (Corning Inc., Corning, NY, USA). To monitor cell density and viability, a Vi-CELL^TM^ cell viability analyzer (XR Model, Beckman Coulter Life Sciences, Indianapolis, IN, USA) was used routinely and all cells were maintained over 99% viability during culturing.

### 2.3. Plasmids and Plasmid Preparation

Plasmid DNAs encoding antibody heavy chain (HC) or light chains (LC) were in a cytomegalovirus (CMV)-promoter-based DNA expression vector pTT5. cDNA clones encoding human p21 (pCMV3-p21, Ref Seq number NM_000389.3, CAT#HG11108-UT) and p27 (pCMV3-p27, Ref Seq number NM_004064.3, CAT #HG11109-UT) were purchased from SinoBiological (Wayne, PA, USA). All plasmids carry the ampicillin-resistant gene. To prepare plasmid DNA for cell transfection, plasmid DNAs were transformed into OneShot^TM^ Top10 chemically competent *E.coli* (Thermo Fisher, CAT#C404003), then expanded into 1 L terrific broth medium with 50 µg/mL carbenicillin from single colonies at 37 °C with 210 rpm in New Brunswick Innova TM44 incubator shaker (Eppendorf AG, Hamburg, Germany) for overnight culturing. The plasmid DNAs were purified with GenElute^TM^ HP Select Plasmid Gigaprep Kit (Sigma-Aldrich, CAT#NA0800-1KT). DNA concentrations were determined by UV-spectrophotometer *Nanodrop One^TM^* (Thermo Fisher) and DNA sequences were confirmed with Sanger DNA sequencing.

### 2.4. Transient Transfection Procedures for Expi293F^TM^ and ExpiCHO-S^TM^

The ExpiFectamine™ 293 Transfection Kit and ExpiFectamine^TM^ CHO Transfection Kit were utilized according to the manufacturer’s instructions with modifications. The basic transfection procedures with ExpiFectamine^TM^ were described previously [13]. Essentially, the total DNA concentration used for transfection was 1.0 µg/mL of cell culture. ExpiFectamine™ 293 was used as 2.7 µL/mL of cell culture and ExpiFectamine^TM^ CHO was as 3.2 µL/mL. Both the DNAs and the transfection reagents were first diluted in Opti-MEM^TM^ medium (Thermo Fisher, CAT#31985070) prior to the mixing incubation at room temperature (15 min for ExpiFectamine™ 293 and 2.5 min for ExpiFectamine™ CHO). The DNA/ExpiFectamine^TM^ complexes were added to either Expi293F^TM^ at 3.0 × 10^6^/mL of viable cell density (VCD) or ExpiCHO-S^TM^ at 6.0 × 10^6^/mL VCD with >99% viability. 16 h post-transfection, 5 mL/L of enhancer-1 and 50 mL/L of enhancer-2 were added to the Expi293F^TM^ culture, whereas 6 mL/L of ExpiFectamine^TM^ CHO enhancer and 200 mL/L of ExpiCHO Feed were added to the ExpiCHO-S^TM^ culture. For ExpiCHO-S^TM^, the cell culture was shifted to 32 °C and 80 rpm on the same day, with an additional 120 mL/L ExpiCHO Feed at 96 h post-transfection. The conditioned medium (CM) for Expi293F^TM^ was harvested at 120 h post-transfection and the CM for ExpiCHO-S^TM^ was harvested at 168 h post-transfection.

For PEI-mediated Expi293F^TM^ transfection, 8 mg/L of PEI prediluted into the Opti-MEM^TM^ medium was mixed with 1 mg/L DNAs prediluted into the Opti-MEM^TM^ medium. The DNA-PEI complexes formed after a 15-min-incubation at room temperature were then added to the Expi293F^TM^ culture at 3.0 × 10^6^/mL VCD. 16 h post-transfection, 25 mL/L of Tryptone N1 (20%) and 8 mL/L of VPA (500 mM) were added to the culture. The CM was harvested at 120 h post-transfection. For the PEI-based ExpiCHO-S^TM^ transfection, the procedures were previously described [29]. In essence, 7 mg/L of PEI was mixed with 2 mg/L DNAs for a 2.5-min-incubation at room temperature, prior to the mixture inoculation to the ExpiCHO-S^TM^ culture; 16 h post-transfection, 20 mL/L of sodium butyrate (4 mM), 10 mL/L of DMSO, and 100 mL/L of CHO CD EfficientFeed^TM^-B medium (Thermo Fisher, CAT#A10240-01) were added. Then, the cell culture was shifted to 32 °C and 80 rpm on the same day, with an additional 100 mL/L CHO CD EfficientFeed^TM^-B at 96 h post-transfection. The CM was harvested at 168 h post-transfection.

### 2.5. Expression Titer Determination and Quality Control for Expressed Antibodies

Titers for expressed antibodies were determined by ÄKTA chromatography system (Cytiva, Marlborough, MA, USA) or Agilent HPLC-1260 (Agilent Technologies, Santa Clara, CA) with HiTrap^TM^ MabSelectSuRe^TM^ 1 mL column (Cytiva, CAT#11003493). The wash buffer was phosphate-buffered saline (PBS, 137 mM NaCl, 2.7 mM KCl, 8.1 mM Na_2_HPO_4_, 2.7 mM KH_2_PO_4_, pH 7.2) and elution buffer was 150 mM Glycine, 40 mM NaCl, pH 3.5. The eluted antibody solution was neutralized with 10% of 2M HEPES, pH 8.0, and subjected to a size exclusion chromatography analysis with Superdex200 10/300 GL (Cytiva, CAT# GE28-9909-44) with 40 min at 0.75 mL/min flow rate in Agilent HPLC-1200. The antibody eluants were also analyzed by SDS-PAGE and visualized by coomassie blue staining.

### 2.6. Liquid Chromatography (LC)–Mass Spectrometry (MS) Protein Sample Preparation

Protein samples were initially diluted to 1.0 mg/mL in PBS (calcium/magnesium-free, pH 7.2) and deglycosylated using recombinant PNGaseF (New England BioLabs, Ipswich, MA, USA). Dithiothreitol (Thermo Fisher Scientific) was added to the reduced samples at a final concentration of 50 mM. Intact and reduced samples were incubated for 2 h at 37 °C and then diluted to 0.25 mg/mL using 0.1% formic acid in water (Thermo Fisher Scientific).

### 2.7. LC-MS Instrument Methods

Prepared protein samples were processed using an ACQUITY UPLC I-Class PLUS System (Waters) and Waters UNIFI software (version 1.9.4.053). Each sample was injected at 250 ng and separated over a BioResolve mAb Polyphenyl reverse-phase column (450 Å, 2.7 µm) held at 65 °C. Mobile phases A and B were LC-MS Grade 0.1% formic acid in Water and LC-MS Grade 0.1% formic acid in acetonitrile (Thermo Fisher Scientific), respectively. Each run was performed at 0.400 mL/min with the following gradient % B settings: 0 min 5% B, 3 min 5% B, 6 min 85% B, 7 min 85% B, 7.10 min 95% B, 9 min 95% B, 9.10 min 5% B, 10 min 5% B. The ACQUITY RDa Detector for mass spectrometry was set to full scan and positive ion mode with high mass range scanning (400–4000 *m/z*). The cone voltage for intact analysis was 70 V and 45 V for reduced analysis. The data were processed using Maximum Entropy settings.

### 2.8. Dynamic Light Scattering

Dynamic Light Scattering (DLS) studies were performed using a Malvern Zetasizer UltraRed DLS instrument (Malvern Panalytical Ltd., Malvern, UK) and ZS XPLORER (v 3.1.0.64) software. Transfection reagents and transfection reagent-DNA complexes were mixed at room temperature and transferred (2 mL) to disposable cuvettes immediately prior to DLS measurement. Complex sizing was monitored at 25 °C for 30–40 min, plotting Z-average diameter (nm) versus time (min).

## 3. Results

### 3.1. The Volume of Opti-MEM^TM^ Dilution Buffer for DNAs and Transfection Reagents Was Critical for the TGE Protein Productivity in Expi293F^TM^ and ExpiCHO-S^TM^ Cells

**Figure 1:**
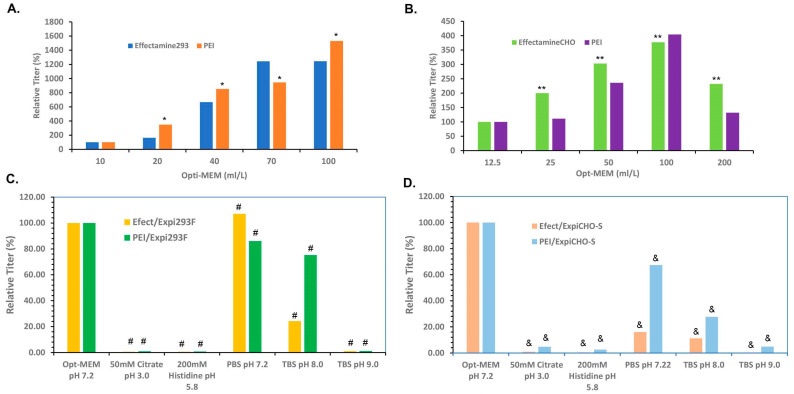
Optimizing the dilution buffer processes for the DNA complexation with ExpiFectamine™ and PEI for Expi293F^TM^ and ExpiCHO-S^TM^. As described in Section 2.4, the target DNAs and the transfection reagents were diluted separately in different volumes of Opti-MEM^TM^ (mL/L cell culture volume per tube); prior to the mixing of the two components for the complexation formation. For Expi293F^TM^, the expression titers were determined five days post-transfection. For ExpiCHO-S^TM^, the expression titers were measured seven days post-transfection. (**A**) The Opti-MEM^TM^ medium dilution conditions for the DNA complexation with ExpiFectamine™ 293 and PEI (*, *p* < 0.05) in Expi293F^TM^ for antibody-A. The titers obtained under the conditions of 10 mL/L per tube were set as 100%. (**B**) The Opti-MEM^TM^ medium dilution conditions for the DNA complexation with ExpiFectamine™ CHO (**, *p* < 0.05) and PEI in ExpiCHO-S^TM^ for antibody-A. The titers obtained under the conditions of 10 mL/L per tube were set as 100%. (**C**) The pH effects of the dilution buffers for the DNA complexation with ExpiFectamine™ 293 (#, *p* < 0.05) and PEI (#, *p* < 0.05) in Expi293F^TM^ for antibody-A. The Opti-MEM^TM^ medium and other indicated dilution buffers in different pHs were used for diluting the DNAs and the transfection agents in 100 mL/L per tube. The titers obtained under the conditions of Opti-MEM^TM^ medium were set as 100%. (**D**) The pH effects of the dilution buffers for the DNA complexation with ExpiFectamine™-CHO (&, *p* < 0.05) and PEI (&, *p* < 0.05) in ExpiCHO-S^TM^ for antibody-A. The titers obtained under the conditions of Opti-MEM^TM^ medium were set as 100%.

It is reasonable to hypothesize that each experimental step in the TGE processes can have an influence on the final protein productivity. The DNA/transfection reagent complex formation step is the beginning part of the transfection process, and we first sought to optimize this reaction step. Prior to the mixing and incubation, both plasmid DNAs and transfection reagents are typically diluted into Opti-MEM^TM^ medium in a separate tube. This manipulation was originally used for enabling easy mixing, as the volumes of DNAs and transfection agents used are normally too small to be mixed thoroughly. In Figure 1A, Opti-MEM^TM^ volumes from 10 mL/L transfection scale to 100 mL/L were used each for the dilution processes of plasmid DNAs encoding antibody-A with either ExpiFectamine™ 293 or PEI for the transfection in Expi293F^TM^ cells. In contrast to the common assumption that the higher the concentrations of the reagents, the better transfection efficiency, the expression titers at 10 mL/L Opti-MEM^TM^ were the lowest for both ExpiFectamine™ 293 and PEI. The expression titers in Expi293F^TM^ cells continued to increase drastically up to Opti-MEM^TM^ dilution at 70 mL/L and then leveled off at 100 mL/L. As shown in Figure 1B, a similar volume effect was observed for both ExpiFectamine™CHO and PEI-mediated transfection in ExpiCHO-S^TM^ cells. The titers continued to increase up to the Opti-MEM^TM^ dilution at 80 mL/L and then decreased at 160 mL/L. These data together indicate that the proper dilution of plasmid DNAs and the transfection reagents prior to the complexation can significantly increase the titers of transient expression in both Expi293F^TM^ and ExpiCHO-S^TM^ cells.

To investigate the pH effect on the DNA/transfection reagents formation, 50 mM Citrate, pH 3.0, 200 mM Histidine pH 5.8, PBS pH 7.2, Tris Buffered Saline (TBS, pH 8.0), and TBS pH 9.0 were used for buffer dilution, along with Opti-MEM^TM^ as the control. As shown in Figure 1C, PBS pH 7.2 was interchangeable with Opti-MEM^TM^ for ExpiFectamine™ 293-mediated transfection in Expi293F^TM^ cells, whereas the transfection was drastically decreased in TBS pH 8.0 and there was nearly no expression in 50 mM Citrate, pH 3.0, 200 mM Histidine pH 5.8, and TBS pH 9.0. The PEI-mediated transfection in Expi293F^TM^ cells retained substantial expression at PBS pH 7.2 and TBS pH 8.0, with no expression in other pHs. As shown in Figure 1D, reasonable expression was detected in ExpiCHO-S^TM^ with PBS pH 7.2 in the PEI-mediated transfection, similar to that in Expi293F^TM^ cells. However, for the ExpiFectamine™CHO-mediated transfection, no buffer other than Opti-MEM^TM^, not even PBS pH 7.2, achieved any reasonable expression in ExpiCHO-S^TM^ cells. These data together reveal that the complexation processes for DNAs/transfection reagents in both Expi293F^TM^ and ExpiCHO-S^TM^ cells are sensitive to the changes in pH and that Opti-MEM^TM^ remains the only dilution buffer for ExpiCHO-S^TM^ whereas PBS can be used for the ExpiFectamine™ 293-mediated transfection in Expi293F^TM^ cells.

### 3.2. Expi293F^TM^ and ExpiCHO-S^TM^ Cells Responded Differently to PEI/DNA Ratios

**Figure 2:**
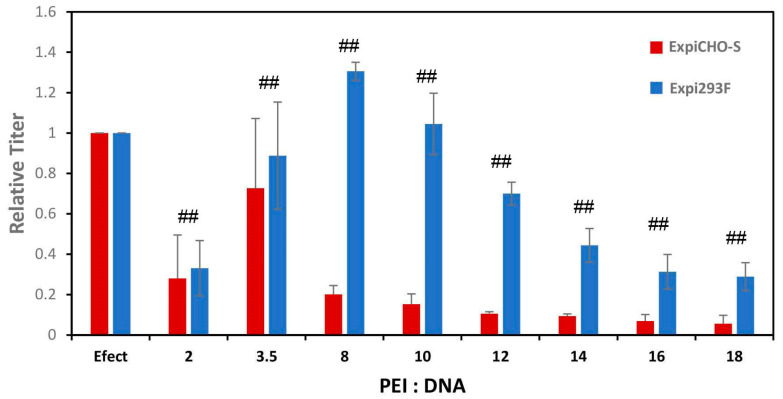
Optimizing the ratios between PEI and DNA in Expi293F^TM^ and ExpiCHO-S^TM^. As described in Materials & Methods, the target DNAs encoding antibody-A were incubated with PEI in various ratios and transfected into Expi293F^TM^ and ExpiCHO-S^TM^ cells. For Expi293F^TM^, the expression titers were determined five days post-transfection. For ExpiCHO-S^TM^, the expression titers were measured seven days post-transfection. The titers obtained for those with ExpiFectamine™ were set as 100% (*n* = 4 ± S.D., ##, *p* < 0.05)).

PEI is a cost-effective cationic polymer transfection reagent alternative to the commercial cationic lipid transfection reagent such as ExpiFectamine™ for transient expression, even though its transfection efficiency is usually lower than that of cationic lipids [30]. In order to further optimize the PEI-mediated expression protocols under the new Opti-MEM^TM^ dilution condition, different ratios of PEI to plasmid DNAs were titrated for the transfection in Expi293F^TM^ and ExpiCHO-S^TM^ cells with antibody-A. As shown in Figure 2, the best PEI/DNA ratio for Expi293F^TM^ was 8. Under both this ratio condition and the ratio condition of 10, the PEI-mediated expression was as good as the ExpiFectamine™293-mediated expression. When the PEI/DNA ratio increased over 10, the expression titer decreased gradually. For ExpiCHO-S^TM^, the best PEI/DNA ratio was 3.5. Under this condition, the titer for the PEI-mediated expression was about 80% of the ExpiFectamine™CHO-mediated expression. In contrast to those in Expi293F^TM^, the expression titer in ExpiCHO-S^TM^ decreased sharply as the PEI/DNA ratio increased over 3.5. These findings indicate that PEI can substitute ExpiFectamine™ better for Expi293F^TM^ than for ExpiCHO-S^TM^ cells and that these two cell types responded differently to the PEI/DNA ratios.

### 3.3. ExpiCHO-S^TM^ Cells Preferred a Shorter Complex Formation Time Than That of Expi293F^TM^ Cells

**Figure 3:**
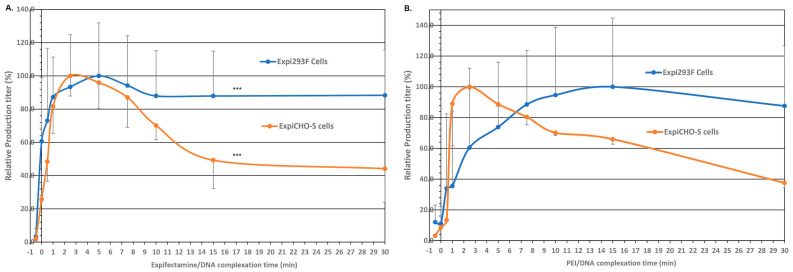
Optimizing the DNA complex formation time in Expi293F^TM^ and ExpiCHO-S^TM^ with ExpiFectamine™ and PEI. As described in Section 2.4, the target DNAs encoding antibody-A were incubated with either ExpiFectamine™ (Panel **A**) or PEI (Panel **B**) for various time points (−0.5 min: the transfection reagents and the DNAs were directly added into the cell culture without mixing; 0 min: the transfection reagents and the DNAs were mixed, but without incubation prior to the addition to cell culture; incubation time after mixing: 0.5 min, 1 min, 2.5 min, 5 min, 10 min, 15 min, and 30 min), and transfected into Expi293F^TM^ and ExpiCHO-S^TM^ cells. The highest titers with each transfection reagent in each cell host were set as 100% (*n* = 3 ± S.D., ***, *p* < 0.05).

Complex formation time for DNA and transfection reagents is another important parameter for the transient transfection process. In order to ascertain the optimal DNA complex formation time for either ExpiFectamine™ or PEI, a time course study in Expi293F^TM^ and ExpiCHO-S^TM^ cells was conducted for the effect on expression titers. As shown in Figure 3A, there was little expression in Expi293F^TM^ or ExpiCHO-S^TM^ when ExpiFectamine™293 or ExpiFectamine™CHO was added directly to cell culture followed by immediate addition of the plasmid DNA encoding antibody-A (−0.5 min). However, significant expression was detected for both cell hosts when the complexes were added to the culture immediately after the mixing without incubation (0 min). For Expi293F^TM^ cells, the expression titer continued to increase and reached the highest peak at 5 min, then gradually leveled off up to 30 min. In contrast, ExpiCHO-S^TM^’s titer raised sharply and reached the highest peak at 2.5 min. After that, the titer decreased sharply up to 15 min and then leveled off to 30 min.

As shown in Figure 3B, the time course study for PEI in Expi293F^TM^ and ExpiCHO-S^TM^ cells revealed a pattern similar to those of ExpiFectamine™. There was little expression in Expi293F^TM^ or ExpiCHO-S^TM^ when PEI was added directly to cell culture followed immediately with the addition of plasmid DNAs (−0.5 min). Different from that of ExpiFectamine™ at 0 min (Figure 3A), the expression levels in both cell lines were still low when the complexes were added to the culture immediately after the mixing without incubation (0 min) (Figure 3B). Then for Expi293F^TM^, the titer continued to increase as the incubation time increased. It reached its highest peak at 15 min and leveled off up to 30 min. In contrast, ExpiCHO-S^TM^ cells’ titer rose sharply, reached the highest peak at 2.5 min, and then decreased significantly afterward up to 30 min. These observations on PEI mimicked the patterns of ExpiFectamine™ in Expi293F^TM^ and ExpiCHO-S^TM^ cells, respectively. These data together indicate that for both ExpiFectamine™ and PEI, ExpiCHO-S^TM^ cells expressed better with a shorter DNA/transfection reagent complex formation time whereas Expi293F^TM^ cells preferred a longer one.

### 3.4. The Size of the DNA Complexes with Either ExpiFectamine™CHO or PEI Increased as a Function of the Incubation Time

**Figure 4:**
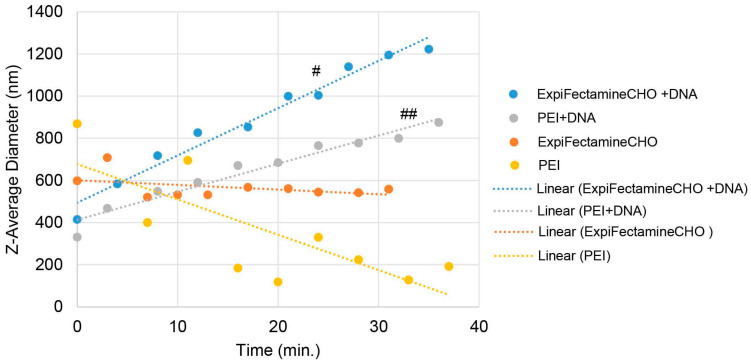
Size measurement of the DNA: ExpiFectamine™CHO or PEI complex with DLS. DLS measurements for size determination were described in Section 2.8. ExpiFectamine™CHO alone, or PEI alone, or in complex with the target DNAs encoding antibody-A (DNA:PEI = 1:3.5; DNA: ExpiFectamine™CHO = 1 µg:3.2 µL) performed in Opti-MEM^TM^ medium (#, *p* < 0.05, ##, *p* < 0.05).

Various complex formation times for the DNA/transfection reagent might have an impact on the size of the complexes, which might be attributed to the transfection efficiency differences observed between Expi293F^TM^ and ExpiCHO-S^TM^ cells. To determine whether or not the size of the DNA/transfection agent complexes changes during the incubation time, we set out to perform the DLS analysis. As shown in Figure 4, the DNA complex with ExpiFectamine™CHO increased with a Z-average diameter ranging from 600 nM to 1200 nM, whereas the diameter for the ExpiFectamine™CHO alone remained constant. Similarly but to a lesser extent, the DNA complex with PEI also continued to increase over time with a Z-average diameter ranging from 400 nM to 800 nM, while the diameter for the PEI alone scattered dynamically. Yet the DNA condensation by ExpiFectamine™293 did not result in dominant homogeneous complexes such as those of ExpiFectamine™CHO and PEI. These data together indicate that the sizes for the DNA complexes with ExpiFectamine™CHO and PEI increased over time, whereas those for the ExpiFectamine™293 DNA complexes scattered dynamically.

### 3.5. Expi293F^TM^ and ExpiCHO-S^TM^ Cells Responded Differently to the Endocytosis Blockers

**Figure 5:**
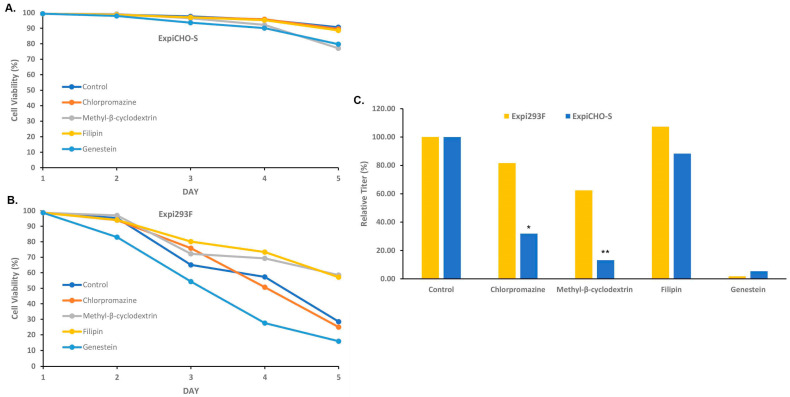
Expi293F^TM^ and ExpiCHO-S^TM^ cells responded differently to the endocytosis blockers. As described in Section 2.4, various endocytosis blockers were added to the cell culture of Expi293F^TM^ and ExpiCHO-S^TM^ prior to the transfection of the target DNAs encoding antibody-A complexed with ExpiFectamine™. Cell viability in ExpiCHO-S^TM^ (pane **A**), and Expi293F^TM^ (panel **B**) as well as expression titers (panel **C**) were determined. The titers for the mock-treated control in each cell host were set as 100% (*, *p* < 0.05, **, *p* < 0.05).

Different complexation time preferences by Expi293F^TM^ and ExpiCHO-S^TM^, as well as the different size changes in the complexes with ExpiFectamine™CHO and ExpiFectamine™293, imply that Expi293F^TM^ and ExpiCHO-S^TM^ cells might utilize different DNA uptake pathways. To investigate how the endocytosis blockers affect the TGE protein productivity in these two cell lines, a pharmacological experiment was performed. The Expi293F^TM^ and ExpiCHO-S^TM^ cells were pretreated with endocytic pathway inhibitors and then transfected with plasmid DNAs encoding antibody-A three hours later. Four types of endocytosis inhibitors were tested: 100 µM chlorpromazine which is known to disrupt clathrin assembly and block the clathrin-dependent endocytosis [31], 1 mM methyl-β-cyclodextrin and 5 µg/mL Filipin that are inhibitors of caveolae formation through cholesterol binding and depletion [32,33], and 200 µM Genestein, a tyrosine kinase inhibitor that can also inhibit caveolar uptake [34,35]. As shown in Figure 5A, ExpiCHO-S^TM^ cells maintained high viability after the treatments of these inhibitors, whereas the cell viability of Expi293F^TM^ decreased over time in all conditions (Figure 5B). Interestingly, protein expression in ExpiCHO-S^TM^ was drastically inhibited by the treatments of methyl-β-cyclodextrin and Genestein, substantially by that of chlorpromazine (Figure 5C). This suggests that both the caveolae and clathrin-dependent pathways might be involved in the DNA complex uptake in ExpiCHO-S^TM^ cells. In Expi293F^TM^ cells, antibody expression was completely eliminated by the treatment of Genestein, but not affected by other treatments, suggesting that the caveolae pathway is the major route in Expi293F^TM^ cells. These data together indicate that Expi293F^TM^ and ExpiCHO-S^TM^ cells respond differently to the endocytosis blockers for the TGE process.

### 3.6. Expi293F^TM^ and ExpiCHO-S^TM^ Cells Responded Differently to the Cotransfection of Cell Cycle Inhibitor p21 and p27

**Figure 6:**
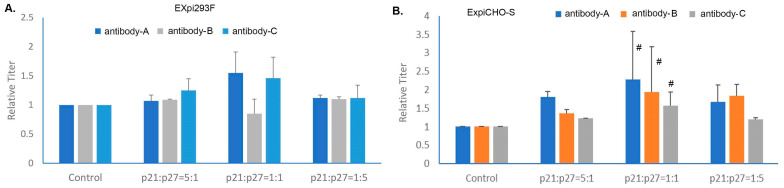
Co-transfecting the genes encoding cell cycle inhibitor p21 and p27 enhanced transient expression in Expi293F^TM^ and ExpiCHO-S^TM^ cells, with a bigger effect in ExpiCHO-S^TM^. As described in Section 2.4, the target DNAs encoding antibody-A, antibody-B, and antibody C complexed with ExpiFectamine™ along with different amounts of plasmid DNAs encoding p21/p27 were transfected into Expi293F^TM^ (Panel **A**) and ExpiCHO-S^TM^ (Panel **B**) cells. The control titers without p21/p27 DNAs were set as 100% (*n* = 3 ± S.D, #, *p* < 0.05).

Inducing controlled proliferation is a strategy for improving protein yield, and co-transfection with cell cycle inhibitors p21/p27 has been previously reported to enhance TGE in HEK293 and CHO-K1 cells [27,36,37]. We sought to determine whether or not this cotransfection strategy can improve TGE efficiency in Expi293F^TM^ and ExpiCHO-S^TM^ cells. As shown in Figure 6, antibodies-A, B, and C along with three different ratios of p21:p27 (5:1, 1:1, 1:5) have been tested. At a 1:1 ratio, p21:p27 enhanced the expression of antibody-A in Expi293F^TM^ by 50% and in ExpiCHO-S^TM^ by 2.5 folds. It also enhanced antibody-C expression in Expi293F^TM^ cells by 40%, and in ExpiCHO-S^TM^ antibody-B by 2 folds and antibody-C by 1.5 fold. At a 5:1 ratio, antibody-C expression was enhanced in Expi293F^TM^ by 20%, and antibody-A expression was enhanced in ExpiCHO-S^TM^ by 2 folds. At 1:5 ratio, expression of antibody-A and antibody-B were enhanced in ExpiCHO-S^TM^ by 40% and 60%, respectively, while no enhancement effect was detected in Expi293F^TM^ cells. These results together suggest that ExpiCHO-S^TM^ seemed to respond better than Expi293F^TM^ to the cotransfection of p21/p27 when transiently producing various antibodies.

### 3.7. DMSO Could Enhance More Transient Expression in ExpiCHO-S^TM^ Cells Than in Expi293F^TM^ Cells as the Concentration Increased

**Figure 7:**
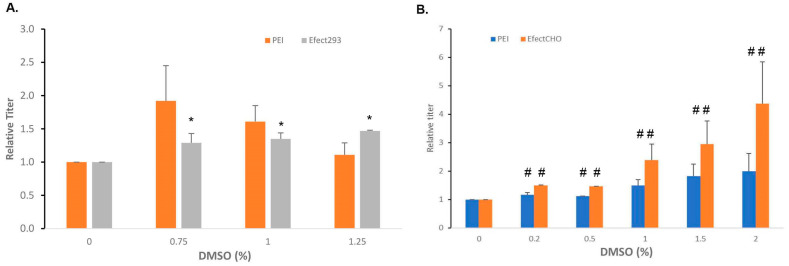
DMSO with increased concentrations could enhance more transient expression in ExpiCHO-S^TM^ cells than in Expi293F^TM^ cells. As described in Section 2.4, Expi293F^TM^ (Panel **A**) and ExpiCHO-S^TM^ (Panel **B**) cells were pretreated with different concentrations of DMSO prior to the DNA transfection for antibody-A. The control titers without DMSO treatment were set as 100% (*n* = 3 ± S.D., *, *p* < 0.05, #, *p* < 0.05).

Adding expression enhancers during cell culture is an important step for the TGE processes. We tested several reported chemicals in the Expi293F^TM^ and ExpiCHO-S^TM^ systems. The addition of lithium acetate (LiOAc) was reported to enhance transient expression in transient CHO cells [10]. Adding potent CDK4/6 inhibitors such as Palbociclib [38] to the cell culture did not result in a meaningful expression titer enhancement at the concentrations of 1–40 µM . However, when the DMSO effect [10,39] was explored in the Expi293F^TM^ and ExpiCHO-S^TM^ cells, significant beneficial results were detected in ExpiCHO-S^TM^ cells but not in Expi293F^TM^ cells. As shown in Figure 7, 0.2–2% of DMSO, added to the cell culture three hours before the transfection, significantly increased expression in ExpiCHO-S^TM^ cells, with the highest effect seen in the concentration of 2%. The enhancement effect was detected in both ExpiFectamine™CHO- and PEI-mediated transfections, with a bigger improvement with ExpiFectamine™CHO. For Expi293F^TM^ cells, 0.75% DMSO enhanced the PEI-mediated expression of antibody-A, with less effect at higher concentrations. For the ExpiFectamine™293-mediated transfection, no enhancement was detected. These data indicate that ExpiCHO-S^TM^ cells responded better to the expression-enhancing DMSO activity than Expi293F^TM^ cells.

### 3.8. Expression Preferences for Kappa and Lambda Light Chain Isotype Were Detected in Expi293F^TM^ and ExpiCHO-S^TM^ Cells

**Figure 8:**
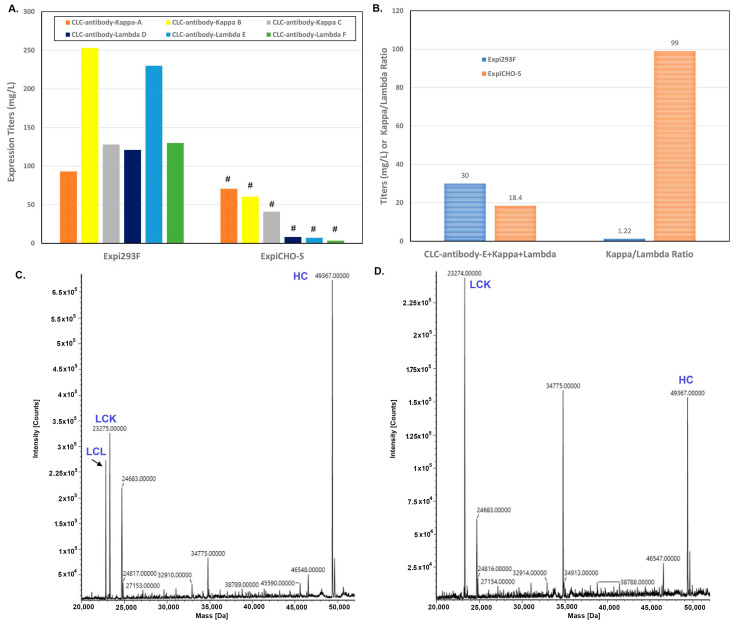
Expression preferences for kappa and lambda light chain isotypes were detected in Expi293F^TM^ and ExpiCHO-S^TM^. As described in Section 2.4, the target DNAs encoding common light chain (CLC) antibodies A, B, and C with a kappa light chain or CLC antibody-D, E, and F with a lambda light chain were transfected into either Expi293F^TM^ or ExpiCHO-S^TM^ cells. (**A**) Expi293F^TM^ produced high titers for CLC antibodies A-C with kappa light chain and for CLC antibodies D-F with a lambda light chain, yet ExpiCHO-S^TM^ only expressed well for CLC antibodies A-C with kappa light chain (#, *p* < 0.05). (**B**) Co-transfecting heavy chain (HC) of CLC antibody E with both kappa (LCK) and lambda (LCL) light chain into either Expi293F^TM^ or ExpiCHO-S^TM^ cells resulted in 1:1 kappa/lambda expression ratio in Expi293F^TM^ cells but 99:1 expression ratio in ExpiCHO-S^TM^ cells. (**C**) Mass spectrometry analysis of the kappa and lambda co-transfection in Expi293F^TM^ cells. (**D**) Mass spectrometry analysis of the kappa and lambda co-transfection in ExpiCHO-S^TM^ cells.

Antibodies contain two types of light chains, kappa and lambda, while their ratios in animals vary. Therapeutic antibody hits could come from different animal sources. For humans, the ratio of kappa and lambda is between 1.7 and 2 with more lambda during early infancy and old age [40]. Murine has a ratio of about 17 [41], whereas the ratios in animals like dogs, horses, and cattle are around 0.1 [42]. There is no report about whether or not Expi293F^TM^ and ExpiCHO-S^TM^ cells express differently the antibodies with different light chain isotypes. In this study, when a set of human antibodies sharing a common light chain (CLC antibodies Kappa A-C and CLC antibodies Lambda D-F) were produced in Expi293F^TM^ and ExpiCHO-S^TM^ cells, we found ExpiCHO-S^TM^ cells produced reasonably good expression titers to the antibodies with a common kappa light chain (CLC antibodies Kappa A–C) but very poorly to the antibodies with a common lambda light chain (CLC antibodies Lambda D–F, Figure 8A). In contrast, Expi293F^TM^ cells produced reasonably well the same set of antibodies with either lambda or kappa light chains (Figure 8A).

To further confirm that there is indeed a kappa light chain expression bias for ExpiCHO-S^TM^ cells, CLC antibody-E with one heavy chain, one kappa light chain, and one lambda light chain were co-transfected into either ExpiCHO-S^TM^ or Expi293F^TM^, respectively. Consistently, the titer in Expi293F^TM^ was significantly higher than that in ExpiCHO-S^TM^ (Figure 8B). The purified antibody protein was then analyzed by mass spectrometry. As shown in Figure 8B–D, Expi293F^TM^-derived CLC antibody-E contained a similar amount of kappa and lambda light chains, whereas ExpiCHO-S^TM^-derived CLC antibody-E only contained kappa light chain with almost no detectable lambda light chain. These results indicate that ExpiCHO-S^TM^ cells can preferably express certain antibodies with kappa light chains over those with lambda light chain, while Expi293F^TM^ cells seems producing similarly well the same set of antibodies with either light chain isotypes.

## 4. Discussion

The TGE technologies in mammalian cells are a powerful tool for the rapid generation of many high-quality therapeutic hits supplied from the ever-evolving antibody discovery platforms, such as display technologies, hybridoma, and single-cell technologies, as well as computational and rational engineering. Yet, the TGE technologies still face major challenges like low production yield and batch-to-batch variation. To improve the TGE production yield and robustness, this study has examined the TGE production processes by investigating the parametric and mechanistic differences between the commercial cell host Expi293F^TM^ and ExpiCHO-S^TM^. While both systems could be optimized by increasing the volume for the complex dilution medium, ExpiCHO-S^TM^ appeared to be more sensitive to the subtle changes in the DNA complex formation process and preferred a much shorter complexation time than Expi293F^TM^. Also, ExpiCHO-S^TM^ was found to be more responsive to the changes in buffers and DNA-to-PEI ratios as well as the treatments of DMSO and cell cycle inhibitors. In addition, this study has revealed that for certain antibodies with common light chains, ExpiCHO-S^TM^ exhibited an expression preference towards those with kappa light-chain isotype over the lambda isotype during the TGE production, whereas Expi293F^TM^ did not seem to have such a preference with the same set of antibodies tested. These interesting observations might reflect the mechanistic differences between the TGE processes in Expi293F^TM^ and ExpiCHO-S^TM^, providing a future direction for optimization.

Several interesting findings from the optimization efforts of this study have been unexpectedly centering on the DNA complex formation step. Firstly, the dilution effects observed on both cationic lipid ExpiFectamine^TM^ and cationic polymer PEI appeared to be counterintuitive. The common sense is that the higher concentration of the plasmid DNAs and the transfection reagents should result from a more efficient transfection and therefore produce a higher production yield. Nonetheless, this study revealed that a further dilution actually gave much-improved expression titers. More dilutions on DNA and transfection reagents imply that smaller sizes of the DNA complexes are formed and preferred by the TGE processes. Secondly, Expi293F^TM^ and ExpiCHO-S^TM^ were found to differ significantly in the DNA complex formation time. ExpiCHO-S^TM^ preferred a much shorter incubation time than Expi293F^TM^, indicating that ExpiCHO-S^TM^ uptakes more efficiently a smaller size of the DNA complexes than Expi293F^TM^. This observation is consistent with the finding that ExpiCHO-S^TM^ and Expi293F^TM^ responded differently to the treatments of endocytosis inhibitors. It has been reported that the particle sizes for the caveolae pathways and the clathrin-dependent pathways are different [34,43].

This study confirms the beneficial effects of DMSO, whereas published potential enhancer LiOAc showed no expression improvement, likely due to the different host systems utilized by the study [25]. DMSO is a small molecule with polar, aprotic, and amphiphilic properties, which has a diverse impact on cells. Low-dose DMSO (0.1–1.5%) treatment can alter membrane lipids and increase membrane permeability [44,45]. Its impacts on DNA conformation, nucleic acid content, protein beta-sheet structure, and cell cycle progression have been reported [45,46]. DMSO can also serve as a chemical chaperon [25,47], and increase cellular mRNA level [39]. The enhancement effects of low-dose DMSO on the transient production in Expi293F^TM^ and ExpiCHO-S^TM^ could be attributed to these above-mentioned mechanisms. Interestingly, this study further uncovered that the ExpiCHO-S^TM^ cells were more responsive to the DMSO treatment than the Expi293F^TM^ cells, suggesting that this effect could be cell-type specific.

This study also validated the beneficial effects of co-transfecting cell cycle inhibitors on the transient production of certain antibodies. P21/p27 are cyclin-dependent kinase inhibitors that regulate cell cycle progression at the G1/S phase [48], and their expression booster effects in FreeStyle^TM^ HEK293F systems were reported previously [27,37]. Our studies showed that co-transfecting p21/p27 at the ratio of 1:1 was significantly better than the published optimized ratio of 1:5 in the Expi293F^TM^ while both ratios worked well in the ExpiCHO-S^TM^ system. Inducing cell growth arrest is a common strategy for improving recombinant protein production, which also includes mild hypothermia by temperature shift [49,50], the addition of histone deacetylase inhibitor (HDACi) [51], and cell cycle kinase chemical inhibitors [52,53]. Since HDACi valproic acid is routinely employed by the Expi293F^TM^ workflow and the 32 °C temperature shift is in the ExpiCHO-S^TM^ workflow, the further enhancement by co-transfecting p21/p27 indicates a synergistic and compatibility effect among these strategies.

It is known that increasing PEI concentration to a peak can improve protein expression but decline afterward. In this study, while the best PEI/DNA ratio was 8 for Expi293F^TM^ and 3.5 for ExpiCHO-S^TM^, the final PEI concentrations for both cell lines were actually similar (8 mg/L and 7 mg/L) due to the twice amounts of DNAs used for ExpiCHO-S^TM^. This PEI concentration range might represent the most tolerated PEI concentration for both cell lines. Increasing PEI concentration can cause cellular toxicities which include endosome membrane rupture and mitochondria dysfunction [54]. This study further revealed that ExpiCHO-S^TM^ seemed to be more sensitive to free PEI than Expi293F^TM^, as its expression titer dropped more sharply when the PEI concentration increased. A larger size of the PEI/DNA complex could also be formed when the PEI/DNA ratio increases. This is consistent with the hypothesis that ExpiCHO-S^TM^ cells prefer a smaller complex size as the expression titer decreased drastically when the complex formation time was over 2.5 min. Expi293F^TM^ might prefer a larger complex size as its expression peaked in 15 min and remained constant for up to 30 min.

Antibody production is known to be affected by factors such as HC:LC ratios [55], variable regions [56], and constant regions [57] of HC and LC. One surprising finding from this study is that ExpiCHO-S^TM^ seems to express much better the Kappa light chain than the lambda isotype for a few antibodies, whereas Expi293F^TM^ has a similar expression preference towards both isotypes. Interestingly, this observation correlates well with the fact that CHO is rodent derived which has much more kappa isotype (95% vs. 5%) than human (60% vs. 40%). Even though CHO does express well many lambda-containing antibodies and variable regions of the light chains also play a role in its expression, there might be species-specific factors that can facilitate the expression of the light-chain isotype. Recently, it was reported that the lambda isotype, but not the kappa light chain, can drastically stimulate the expression of IgMs and IgA2 in Expi293F^TM^ cells [58]. This enhancement does not affect the expression of IgG1 and IgA1. Our data reveal for the first time that CHO cells have an expression preference towards the kappa isotype.

The TGE process in mammalian cells is more complex than expected, as a recent system biology study on transcriptomic analysis of CHO and HEK293 during transient production has revealed their profound differences in the secretary pathway utilization [59]. The findings from this study further support the notion that the TGE processes in CHO and HEK293 might employ additional mechanisms which may include the uptake pathways for the DNA complexes and the expression preference for antibody light-chain isotype. Process improvements through understanding and exploiting these differences need to be evaluated and implemented. With improved titers in the TGE processes, more antibody hits can be generated with a lower culture volume. This should enable a faster, more effective, and economical way to triage new therapeutics designs churning out from machine learning and artificial intelligence, along with the conventional antibody discovery platforms.

## 5. Conclusions

This study discovered the parametric and mechanistic differences in Expi293F^TM^ and ExpiCHO-S^TM^ cells with regard to the TGE processes. The resulting findings generated a further-optimized workflow and parameters (Table 1) for rapid generating antibody candidates, as well as a new direction for improvement.

## Figures and Tables

**Table 1:** Optimized parameters for the TGE processes in Expi293F^TM^ and ExpiCHO-S^TM^ cells.

Parameters	Expi293F^TM^	ExpiCHO-S^TM^
Viable Cell Density	3.0 × 10^6^/mL	6.0 × 10^6^/mL
Complexation Time (ExpiFectamine^TM^)	5 min	2.5 min
Complexation Time (PEI)	15 min	2.5 min
Opti-MEM^TM^ volume (ExpiFectamine^TM^/PEI)	100 mL/L	100 mL/L
1% DMDO (ExpiFectamine^TM^/PEI)	-	+
P27/p21 1:1	-	+
Preferred Light Chain	Lambda/Kappa	Kappa
PBS as dilution buffer	+	-
DNA:PEI ratio	1:8	1:3.5

## Data Availability

Data is contained within the article.
